# Supplementary figures and images for: Complete Genome Sequencing and Comparative Phylogenomics of Nine African Swine Fever Virus (ASFV) Isolates of the Virulent East African p72 Genotype IX without Viral Sequence Enrichment
Source: Viruses. 2024 Sep 14;16(9):1466. doi: 10.3390/v16091466 (PMC11437432; doi:10.3390/v16091466)

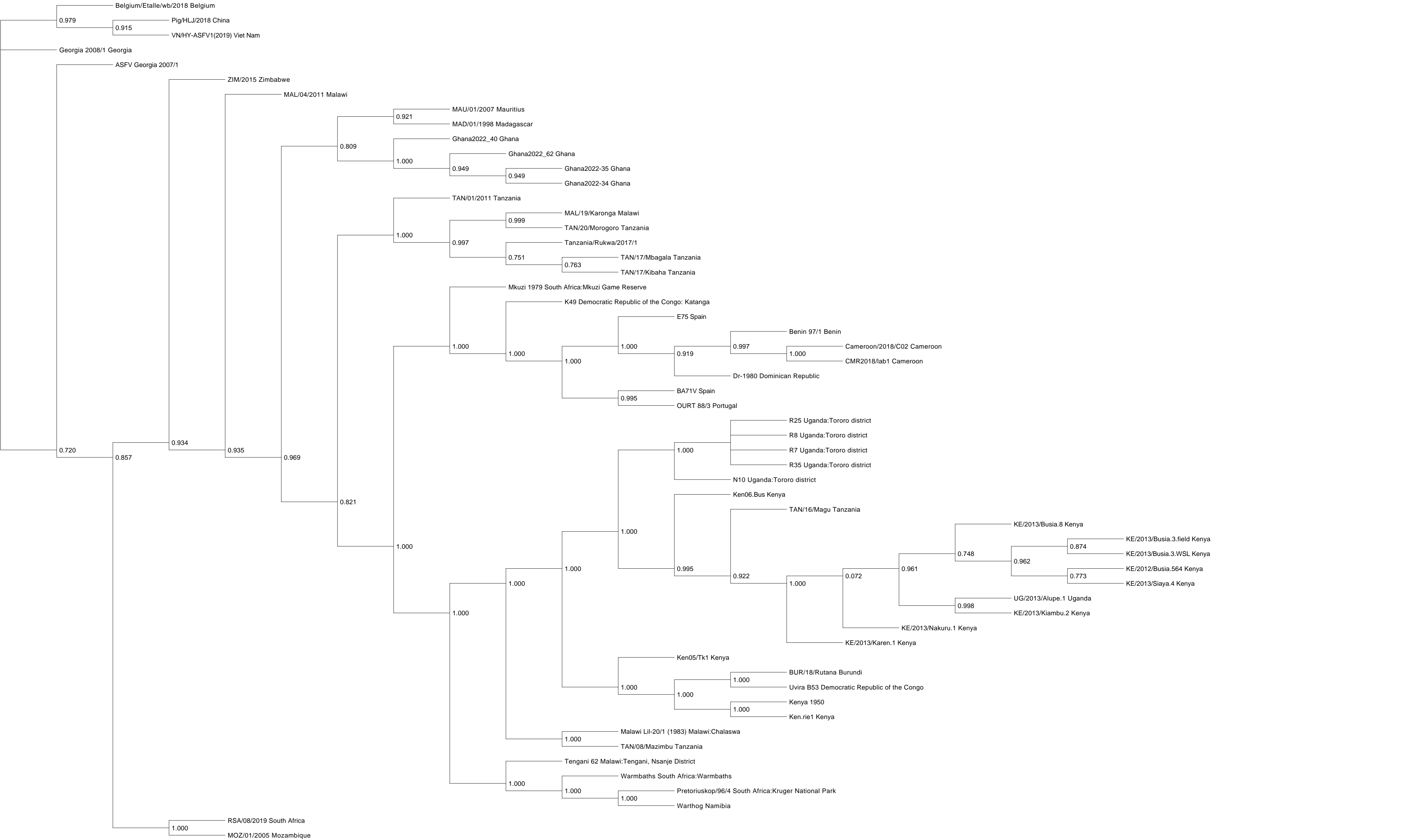

Supplement: Supplementary file 1 [file viruses-16-01466-s001.zip › Supp_FigS3_final_mafft_BMGE_FasttreeGTRGamma_cladogram_with_SH_supports.pdf]

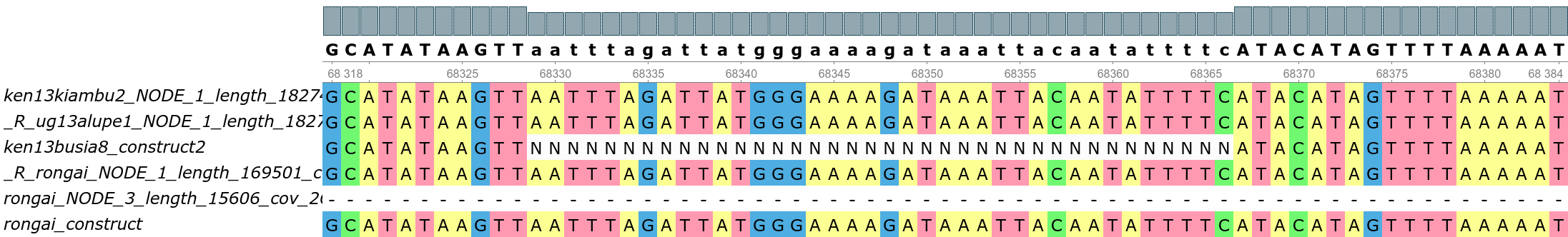

Supplement: Supplementary file 1 [file viruses-16-01466-s001.zip › Supp_Fig_S1_stitching_38N_KE13BUS8_against_others.png]

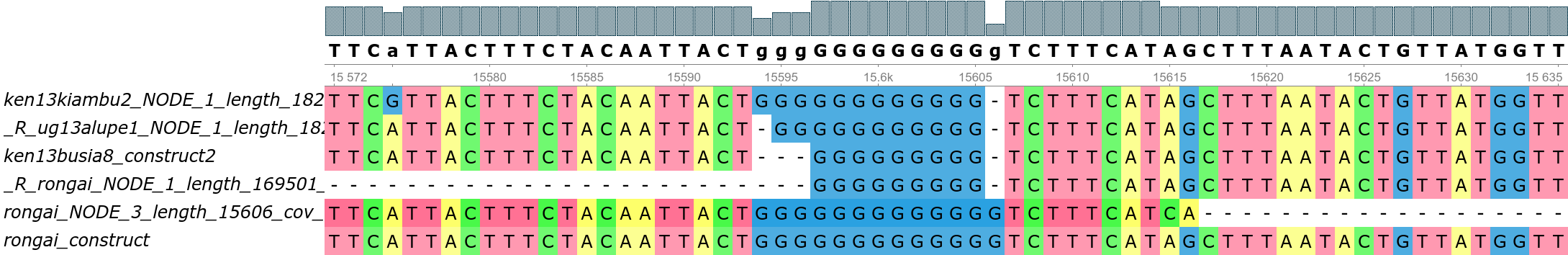

Supplement: Supplementary file 1 [file viruses-16-01466-s001.zip › Supp_Fig_S2_junction_two_contigs_KE2013NAK1.png]
